# Lactation-focused audio relaxation versus standard care for mothers of very preterm infants (the EXPRESS randomised clinical trial)

**DOI:** 10.1038/s41390-024-03577-7

**Published:** 2024-09-25

**Authors:** Ilana Levene, Pollyanna Hardy, Jennifer L. Bell, Christina Cole, Kayleigh Stanbury, Frances O’Brien, Mary Fewtrell, Maria A. Quigley

**Affiliations:** 1 National Perinatal Epidemiology Unit Clinical Trials Unit, Nuffield Department of Population Health, University of Oxford, Oxford, UK; 2 Newborn Care, John Radcliffe Hospital, Oxford, UK; 3 UCL Great Ormond Street Institute of Child Health, Oxford, UK

## Abstract

**Background:**

Mothers of very premature newborns often have low milk supply. Systematic review has shown increased milk quantity with relaxation interventions. We hypothesised that a self-directed audio relaxation and lactation-specific visualisation would increase milk quantity after a very premature birth.

**Methods:**

Unmasked, randomised, controlled trial, recruiting 132 participants in four United Kingdom neonatal units. Eligible women had given birth to one or two infants between 23+0 and 31+6 weeks of gestation. The intervention was a 12-min voice recording including breathing exercises, muscle relaxation and lactation-specific visualisation. Primary outcome was the highest 24-h breastmilk weight expressed on any of day 4, day 14 or day 21 after birth.

**Results:**

Mean birth gestation was 27.8 weeks (SD 2.4), with 26% of participants giving birth under 26 weeks (34/132). Adjusted mean difference in primary outcome was 73.9 g (95% CI −61.7 to 209.5, *p* = 0.28). Spielberger State-Trait Anxiety Index adjusted mean difference was −1.9 (−8.2 to 4.3, *p* = 0.54). The majority of relaxation group participants felt the intervention was relaxing (32/42, 76%).

**Conclusions:**

There was no beneficial effect of this relaxation intervention on milk quantity. Mothers of very premature infants may value relaxation interventions but they are unlikely to have a large effect on milk quantity.

**Impact:**

- This randomised trial did not show a beneficial effect of a self-directed audio relaxation and visualisation on mothers’ own milk quantity expressed after very preterm birth.
- Mothers of very and extremely preterm infants may value relaxation interventions, but they are unlikely to have a large effect on milk quantity.
- Prior systematic review of mixed populations has shown an increase in mothers’ own milk quantity with relaxation interventions. Combining this study with existing meta-analysis could result in a new hypothesis that the lower the gestation at birth, the smaller the impact of relaxation on milk quantity.

## Introduction

An estimated 2.4 million infants^1^ globally are born very preterm (28 to 32 weeks’ gestation) or extremely preterm (less than 28 weeks’ gestation) each year. The ideal nutrition for very preterm infants is their mothers’ own milk (MOM); the greater volume provided, the lower the risk of death, necrotising enterocolitis^2^ and the better the neurodevelopmental outcome.^3,4^

To provide MOM after very preterm birth, mothers often express (mechanically extract milk from the breasts) for weeks to months due to infant oral immaturity. There is a high risk of low milk volume and non-exclusive MOM;^5^ mothers who give birth extremely preterm are at the highest risk.^6,7^ Challenges include abbreviated mammary remodelling in pregnancy, prematurity-related perinatal complications^8^ and the difficulty of establishing lactation by expression. Improving breastfeeding outcomes after very preterm birth is a World Health Organization research priority.^9^

Relaxation is “a state of consciousness characterised by feelings of peace, and release of anxiety and fear”,^10^ which is associated with physiological changes such as reduced heart rate and blood pressure.^10^ Relaxation could potentially improve lactation outcomes by optimising lactogenic hormones (prolactin and oxytocin) via connections with stress hormones such as cortisol.^11,12^ Mental visualisation of milk ejection can trigger milk ejection.^13^ Finally, reduction in stress may improve breastfeeding self-efficacy^14^ and behaviours such as expressing frequency.

Recent meta-analysis reported that relaxation interventions are likely to increase MOM quantity.^15^ Included trials had heterogeneous interventions and populations, and half of the studies in the meta-analysis of milk quantity were assessed as at high risk of bias. Notably, the study recruiting women with the lowest mean birth gestation did not show a significant effect of relaxation (a mindfulness app) on expressed milk quantity (mean difference 132.2 mL, 95% CI −99.3 to 363.7 mL).^16^

Although relaxation interventions have minimal cost to health services, they have significant opportunity cost for mothers. Given that mothers of the most preterm infants are under intense stress,^17,18^ have the highest level of lactation challenge^6,7^ and their infants have the most potential benefit from an increase in MOM provision, it is important to increase the evidence quality for this population.

We hypothesised that a self-directed relaxation and lactation-specific visualisation audio recording would improve MOM quantity and mental health after very preterm birth.

## Methods

### Study design

EXPRESS (Expressing in PREmaturity–Simple interventionS) was an unmasked, parallel group, multi-centre, randomised, controlled trial conducted in four United Kingdom hospital neonatal units. The study was funded by the National Institute for Health and Care Research (NIHR). It was approved by the Bloomsbury Research Ethics Committee, London (21/LO/0279) and registered as ISRCTN 16356650. The trial protocol has been published.^19^

### Participants

Inclusion criteria were birth between 23+0 and 31+6 weeks’ gestation within the previous 3 days; being 18 years or over; intention to express milk for at least 2 weeks; able to give informed consent; and having a device on which to listen to an audio recording. Exclusion criteria were lack of antenatal dating scan and more than two infants. Participants gave written, informed consent on paper or electronically. There were no language constraints specified. Interpretation services were not available, but potential recruits could use their support networks for assistance if needed.

Three English National Health Service trusts were involved, including three tertiary and one local neonatal unit. All sites have hospital-grade breast pumps, a free home loan scheme, dedicated infant feeding and psychological support staff, and provision of donor human milk with varying criteria for use. Two trusts have neonatal UNICEF Baby Friendly Initiative (BFI) UK level three accreditation, suggesting a good level of lactation support. Clinical support was not standardised between sites, as this was considered to represent real-world standard care.

### Randomisation and masking

Participants were randomised (with 1:1 ratio) by site staff using a web-based randomisation system incorporating allocation concealment. The allocation sequence was computer generated by a statistician using randomly permuted stratified blocks of size two and four (using Stata v15.1). Stratification was by recruiting site, gestational age at birth (23+0 to 27+6 or 28+0 to 31+6 weeks) and number of infants (one or two).

### Procedures

The control group received clinical lactation support from neonatal staff. The intervention group received clinical support and a 12-min audio file (Supplementary Audio File 1). They were asked to listen to the recording several times a day while expressing milk. The recording was modified from a soundtrack used for previous studies^20^ in order to ensure that it was appropriate for mothers of sick infants who had never breastfed (rather than the original audience of mothers of healthy breastfeeding infants); the modification process is described elsewhere and involved parent collaborators.^21^ The recording includes breathing exercises, muscle relaxation, and visualisation of milk flow and infant skin-to-skin contact.

Baseline questionnaires were submitted by participants and site staff. On days 4, 14 and 21 after birth, participants recorded each time they expressed milk for 24 h and answered questionnaires, including frequency of listening to the recording.

Participants weighed milk using a portable scale with 0.1 g accuracy (Kabalo). Accuracy with the scale was confirmed by researcher contact between days 4 and 7. Participants responded to SMS (short messaging service) messages at 36 weeks’ postmenstrual age (PMA) and 4 months’ corrected age (CA) to report feeding status. At 36 weeks’ PMA this data was extracted from medical notes if there was no response. Demographics of the potentially eligible population were extracted from routinely entered clinical data.

To maximise efficiency in the available time, participants in the final 22 weeks of recruitment completed the trial at 36 weeks’ PMA. This affects one of the secondary outcomes (exclusive MOM at 4 months’ CA).

The trial contributed to a doctoral thesis and was supported by a Clinical Trials Unit and an independent Trial Steering Committee. An extensive process of Patient, Public Involvement (PPI) is described elsewhere.^21^

### Outcomes

The primary outcome was the highest 24-h expressed MOM weight recorded on any of days 4, 14 or 21. These timepoints were chosen because of prior work showing a close association between early milk yield and longer-term lactation outcomes in the NICU setting.^22,23^ Secondary outcomes were expression of at least 750 g of MOM on any of days 4, 14 or 21; expression rate (milk quantity per minute of expression) at day 21; mental health measures at day 21; any and exclusive MOM at 36 weeks’ PMA and exclusive MOM at 4 months’ CA. Mental health measures were the Spielberger State-Trait Anxiety Index (six item format; STAI-6) and the Post-traumatic stress Checklist for DSM-5 (PCL-5). The STAI-6 was administered at baseline and day 21. The PCL-5 was administered on day 21 only as this checklist applies to experiences in the weeks following a traumatic experience and therefore cannot be administered at baseline. Process indicators were skin-to-skin contact duration, expressing frequency and duration.

### Statistical analysis

The trial was powered to detect an increase in primary outcome from 670 to 825 g (SD 300 g), with 80% power and a two-sided significance level of 0.05. This is a smaller effect size than seen in the meta-analysis.^15^ The control group estimate was informed by local audit data. With 10% attrition expected, the recruitment target was set at 132.

There was a pre-specified statistical analysis plan. For continuous outcomes, this was linear or quantile regression as appropriate, and for binary outcomes log binomial regression or Poisson regression with robust variance estimator if the model failed to converge. Analyses were adjusted for the stratification factors where possible. The STAI score at day 21 was adjusted for baseline score. The primary outcome was adjusted for the associated measurement day (the day that the highest milk weight was recorded).

Participants were analysed in the groups to which they were randomly assigned (the intention to treat the population). Primary analysis was on a complete case basis. Exploratory subgroup analysis used the statistical test of interaction to examine the heterogeneity of treatment effect on the primary outcome by gestational age at birth. Other analyses were summarised by allocation with no comparative statistics, to limit multiple comparisons; statistical inferences were limited to eleven. Exploration of the association of adherence and relaxation perception with the primary outcome was pre-specified, without comparative statistics.

Sensitivity analyses were planned to explore the pattern of missing data for the primary outcome using multiple imputation and pattern mixture models; and by re-defining the parameters of the primary outcome. The four sensitivity analysis redefinitions of the primary outcome were 24-h milk weight on day 21, highest 24-h milk weight on day 14 or 21, excluding records with fewer than four expressing sessions, and excluding records submitted more than 48 h late. The pattern mixture model imputed missing primary outcomes as the average value plus a delta value of −200 g to +200 g, modelling potential bias of missing data by up to 200 g.

Post-hoc random effects meta-regression of milk quantity and gestational age at birth combined the results of this study with neonatal unit studies^16,20,24–27^ included in a recent meta-analysis,^15^ using variance-weighted least squares technique. All study effect estimates for milk quantity were expressed as standardised mean differences. Between-study variance was estimated with residual maximal likelihood. Stata v18 was used for analysis.

The Data Monitoring Committee reviewed one interim report. There were no formal stopping guidelines due to the trial size.

## Results

**Fig. 1:**
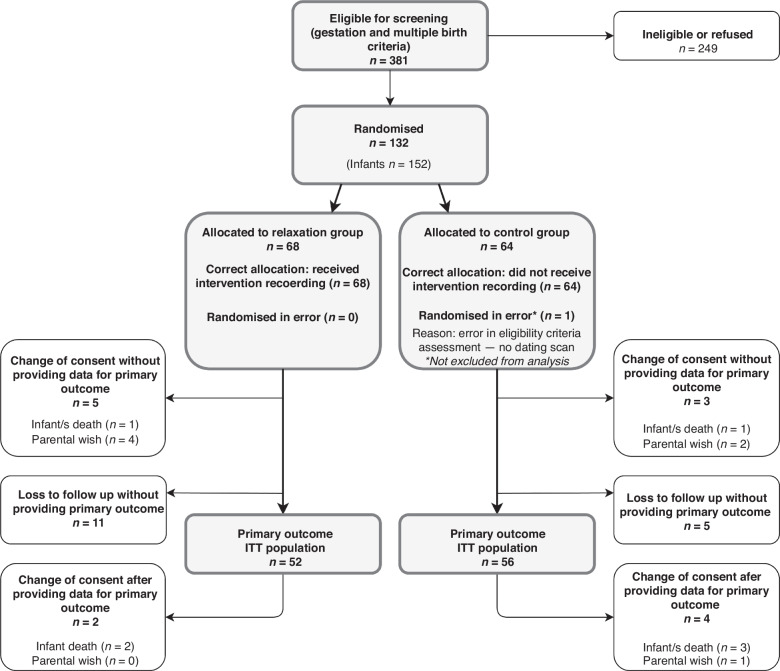
CONSORT flowchart. This demonstrates the flow of participants and data through the trial. ITT intention to treat.

In total, 132 participants were randomised between 2nd August 2021 and 31st October 2022. Figure 1 shows the CONSORT flowchart; 68 participants were allocated to relaxation and 64 to control. Primary outcome data was missing for 16 participants in the relaxation group and 8 in the control group. Data completeness was highest for the primary outcome (82%) and 36 weeks’ PMA feeding assessment (86%).

Table 1 shows baseline characteristics. Mean maternal age (SD) was 32.8 (6.3) years. A quarter of participants gave birth before 26 weeks’ PMA (26%, 34/132). Ethnic background was 18% (22/120) Asian, 18% (21/120) Black and 60% (72/120) White. Overall, 24/131 (18%) lived in the most deprived quintile of English postcodes and 28/131 (21%) in the least deprived quintile.

**Table 1:** Baseline characteristics of the study participants.

	Relaxation group (*n* = 68)	Control group (*n* = 64)
Pregnancy gestation at birth (weeks)^a^, mean (SD)	27.8 (2.4)	27.8 (2.4)
Pregnancy gestation at birth (weeks)^a^, *n* (%)		
23 to <26 weeks	19 (28)	15 (23)
26 to <28 weeks	17 (25)	20 (31)
28 to <30 weeks	15 (22)	14 (22)
30 to <32 weeks	17 (25)	15 (23)
Ethnic background^b^, *n* (%)		
Asian or Asian British	8 (14)	14 (23)
Black, African, Black British or Caribbean	10 (17)	11 (18)
White or White British	38 (66)	34 (55)
Mixed or multiple ethnic groups	0	3 (5)
Other	2 (3)	0
*Missing/prefer not to say*	*10*	*3*
Age (years), mean (SD)	33.1 (6.5)	32.4 (6.2)
(Min to max)	(19 to 55)	(21 to 54)
Index of Multiple Deprivation quintile^c^, *n* (%)		
1 (Most deprived)	15 (22)	9 (14)
2	9 (13)	17 (27)
3	11 (16)	11 (18)
4	15 (22)	16 (25)
5 (Least deprived)	18 (27)	10 (16)
*Missing*	*0*	*1*
Age at leaving full-time education, *n* (%)		
16 years old or less	5 (9)	9 (15)
17 or 18 years old	11 (19)	13 (21)
19 years old or more	42 (72)	40 (65)
*Missing/prefer not to say*	*10*	*2*
Lives with a partner, *n* (%)	54 (92)	54 (87)
*Missing*	*9*	*2*
Current smoker, *n* (%)	4 (7)	4 (7)
*Missing*	*9*	*2*
Time from birth to first expression of milk (hours), median [IQR]	6 [2 to 12]	6 [3 to 12]
(Min to max)	(0 to 96)	(0 to 72)
*Missing*	*9*	*3*
Intention for exclusive breastmilk at discharge home^d^, *n* (%)	41 (70)	44 (71)
*Missing*	*9*	*2*
Spielberger State-Trait Anxiety Index^e^ score at randomisation, median [IQR]	50 [43 to 67]	50 [40 to 60]
(Min to max)	(20 to 80)	(20 to 80)
Score >40	43 (78)	37 (67)
*Missing*	*13*	*9*
Mode of birth, *n* (%)		
Vaginal birth	25 (37)	32 (50)
Caesarean birth	43 (63)	32 (50)
Multiple pregnancy^a^, *n* (%)	11 (16)	9 (14)
Recruiting centre^a^, *n* (%)		
1	39 (57)	38 (59)
2	17 (25)	15 (23)
3	5 (7)	5 (8)
4	7 (10)	6 (9)
Infant born in recruiting centre, *n* (%)	**66 (97)**	**57 (89)**
Postnatal age at randomisation (days), *n* (%)		
Day 0 (day of birth)	2 (3)	5 (8)
Day 1	14 (21)	19 (30)
Day 2	30 (44)	18 (28)
Day 3	22 (32)	22 (34)
Infant ventilated at randomisation (one or both if multiple), *n* (%)	23 (34)	24 (38)
Infant Apgar score at 5 min^f^, *n* (%)		
Median [IQR]	8 [7 to 10]	9 [7 to 10]
<5	4 (6)	1 (2)
*Missing*	*2*	*4*
Primiparous, *n* (%)	37 (60)	37 (60)
*Missing*	*6*	*2*
Multipara only, *N*	25	25
Previous breastmilk feeding experience, *n*/*N* (%)	23 (96)	21 (84)
*Missing*	*1*	*0*
Length of previous breastmilk feeding experience (weeks), median [IQR]	52 [17 to 79]	32 [10 to 52]
Length of previous breastmilk feeding experience, *n* (%)		
<1 week	0 (0)	0 (0)
1 week to <6 weeks	3 (13)	2 (10)
6 weeks to <6 months	5 (22)	8 (38)
6 months to <12 months	4 (17)	5 (24)
≥12 months	11 (48)	6 (29)
*Missing*	*2*	*4*

Missing observations are excluded from percentage denominators.

*SD* standard deviation, *IQR* interquartile range.

^a^Stratification factor.

^b^Categories defined by the United Kingdom Office of National Statistics.

^c^England postcodes are assigned an Index of Multiple Deprivation quintile according to multiple factors associated with area-level deprivation.

^d^Defined as “your baby would be drinking only your breastmilk and no infant formula”.

^e^Six item version transformed to an equivalent score to 20 item original. Score range is 20 to 80, higher score indicates more anxiety.

^f^Lower score if two babies.

Trial participants had similar age, birth gestation, birth mode and rate of multiple pregnancies as the 371 individuals eligible for screening (Supplementary Table 1). In the screening population, 165/341 (48.4%) were primiparous compared with 74/124 (59.7%) of recruits.

On days 4, 14 and 21, relaxation group participants reported listening to the intervention a median of 3 times per day (Supplementary Table 2) and 98% of those who provided adherence data reported ever listening to the recording (51/52). In the control group, 7% (3/44) reported practising non-intervention forms of relaxation daily or more, and 14% (6/44) did so more than once a week but less than daily.

The majority of relaxation group participants liked the recording (25/42, 60%) and felt that it was relaxing (32/42, 76%), whereas 12% disliked it (5/42; Supplementary Table 2). Supplementary Table 3 shows comments by relaxation group participants about the recording. Positive comments related to feeling relaxed, calm or reframing traumatic birth. Negative comments related to monotony, dislike of the recording content or difficulty finding time to listen.

There was no evidence of a difference in the primary outcome of the highest 24-h MOM quantity (adjusted mean difference 73.9 g, 95% CI −61.7 to 209.5 g; Table 2). Fewer participants submitted day 4 and 14 logs in the relaxation group (day 4 relaxation group: 72%, 49/68. Control group: 81%, 52/64; Supplementary Table 2). Similar numbers submitted day 21 logs (Relaxation group: 69%, 47/68. Control group: 67%, 43/64). Thus the primary outcome was formed by day 4 more often in the control group (Relaxation group: 15%, 8/52. Control group: 25%, 14/56). Supplementary Table 4 shows that the pre-specified sensitivity analyses addressing unequal timepoint measurement produced a similar effect size estimate to the adjusted mean difference (which includes adjustment for measurement timepoint).

**Table 2:** Trial outcomes—primary, secondary and process indicators.

	Study recording (*n* = 68)	Control (*n* = 64)	Unadjusted effect estimate (95% CI)	Adjusted^a^ effect estimate (95% CI)	*p*-value
Primary outcome: 24-h expressed milk weight^b^ (grams)					
Mean (SD)	596.7 (433.6)	467.7 (350.2)	MD 129.1 (−20.8 to 278.9)	MD 73.9 (−61.7 to 209.5)	0.28
Median [IQR]	521.4 [254.6 to 902.7]	397.8 [176.6 to 719.2]			
Min to max	(2 to 1926)	(7 to 1274)			
*Missing*	*16*	*8*			
Secondary outcomes					
Expressing ≥750 g milk in 24 h^b^, *n* (%)	18 (35)	12 (21)	RR 1.62 (0.86 to 3.02)	RR 1.43 (0.77 to 2.66)	0.26
*Missing*	*16*	*8*			
Rate of milk expressed day 21 (g/min), median [IQR]	3.7 [2.4 to 6.2]	3.7 [1.8 to 6.2]	Med Diff 0.0 (−1.7 to 1.7)	Med Diff 0.5 (−1.4 to 2.4)	0.59
Min to max	(0 to 13)	(0 to 16)			
*Missing*	*25*	*21*			
STAI^c^ score day 21, mean (SD)	48.3 (15.8)	47.3 (15.9)	MD 1.1 (−5.8 to 7.9)	MD −1.9 (−8.2 to 4.3)	0.54
Min to max	(20 to 80)	(20 to 73)			
Score >40, *n* (%)	25 (58)	23 (56)			
*Missing*	*25*	*23*			
PCL-5^d^ score day 21, median [IQR]	16.5 [9.5 to 24.5]	18 [9 to 32.5]	Med Diff −2.0 (−11.7 to 7.7)	Med Diff −1.4 (−11.8 to 9.1)	0.80
Min to max	(0 to 61)	(0 to 66)			
Score >33, *n* (%)	6 (14)	10 (23)			
*Missing*	*24*	*20*			
Exclusive breastmilk^e^ 36 weeks’ PMA, *n* (%)	37 (64)	37 (67)	RR 0.95 (0.72 to 1.24)	RR 0.96 (0.74 to 1.24)	0.75
*Missing*	*10*	*9*			
Exclusive breastmilk^e^ 4 months’ corrected age, *n* (%)	4 (15)	4 (13)	RR 1.07 (0.29 to 3.89)	RR 1.10 (0.30 to 4.01)	0.89
Not eligible for assessment^f^	25	22			
*Missing*	*16*	*12*			
Any breastmilk^g^ 36 weeks’ PMA, *n* (%)	51 (88)	47 (85)	RR 1.03 (0.89 to 1.19)	RR 1.03 (0.90 to 1.19)	0.67
*Missing*	*10*	*9*			
Process indicators (Day 21):					
Time spent in skin-to-skin contact (hours), mean (SD)	2.2 (1.9)	2.0 (1.8)	Med Diff 0.0 (−1.2 to 1.2)	Med Diff 0.0 (−0.9 to 0.9)	1.00
Median [IQR]	2 [0.8 to 3]	2 [1 to 3]			
Min to max	(0 to 8)	(0 to 9)			
*Missing*	*24*	*23*			
Expressing episodes in 24 h^h^, mean (SD)	5.6 (2.4)	5.9 (1.8)	MD −0.3 (−1.2 to 0.6)	MD −0.3 (−1.1 to 0.6)	0.53
Median [IQR]	6 [5 to 7]	6 [5 to 7]			
Min to max	(0 to 11)	(1 to 8)			
No longer lactating	3 (6.7)	0 (0.0)			
Solely breastfeeding	0 (0.0)	0 (0.0)			
*Missing*	*20*	*21*			
Time spent expressing (hours), mean (SD)	151.5 (90.9)	159.3 (84.9)			
Median [IQR]	151.5 [105 to 200]	150 [110 to 195]	Med Diff 3.0 (−36.2 to 42.2)	Med Diff −2.3 (−40.4 to 35.8)	0.90
Min to max	(0 to 500)	(10 to 480)			
*Missing*	*20*	*21*			
Perception of low milk supply, *n* (%)	9 (21)	17 (39)			
*Missing*	*26*	*20*			
Direct breastfeeds^i^, mean (SD)	0.1 (0.4)	0.0 (0.2)			
Median [IQR]	0 [0 to 0]	0 [0 to 0]			
Min to max	(0 to 2)	(0 to 1)			
*Missing*	*40*	*41*			

Missing observations are excluded from percentage denominators.

*PMA* postmenstrual age, *CI* confidence interval, *SD* standard deviation, *IQR* interquartile range, *RR* relative risk, *MD* mean difference, *Med Diff* median difference.

^a^Adjusted for recruiting hospital, gestational age, multiple births and, as noted below for certain outcomes, measurement day and baseline STAI score.

^b^Highest of any/all submitted logs on days 4, 14 or 21. Adjusted for gestational age at birth, recruitment centre, multiple birth and measurement day.

^c^Six item Spielberger State-Trait Anxiety Index (STAI), transformed to an equivalent score to 20 item STAI. Score range is 20 to 80, higher score indicates more anxiety. Adjusted for gestational age at birth, recruitment centre, multiple births and baseline STAI.

^d^Post-traumatic Stress Scale for DSM-5. Score range is 0 to 80, higher score indicates more post-traumatic stress.

^e^Exclusive breastmilk is defined as no infant formula used in the last 24 h. If two babies, then no infant formula in the last 24 h for either baby. Outcome at 4 months’ corrected was not adjusted for multiple births or centre due to collinearity.

^f^These participants were recruited in the latter part of the study, which concluded at 36 weeks’ PMA.

^g^Any breastmilk is defined as any use of breastmilk in the last 24 h, either direct breastfeeding or as expressed milk. If two babies, then any breastmilk for either baby.

^h^An expressing episode is defined as where two breasts are expressed simultaneously or where two breasts are expressed sequentially, with the start time of the second breast expression within 10 min of the end time of the first breast expression or where one breast is expressed alone.

^i^A direct breastfeed is defined as an episode at the breast where the mother felt the baby was sucking and swallowing some milk. If the baby is offered both breasts in a single episode, this is one breastfeed. If the baby stops sucking or swallowing for 30 min or more, then any further feeding is a new breastfeed. If two babies, then the sum of all episodes with both babies.

In subgroup analysis, the adjusted mean difference in the primary outcome was 53.0 g (95% CI −131.6 to 237.6) in participants with extremely preterm infants and 100.0 g (95% CI −99.3 to 299.4) in participants with very preterm infants (Table 3, interaction *p*-value 0.73).

**Table 3:** Exploratory subgroup analyses.

		Relaxation group (*n* = 68)	Control group (*n* = 64)	Adjusted mean difference (95% CI)
**Gestational age at birth**				
24-h milk weight (g)^a^				
23+0 to 27+6	Mean (SD)	585.8 (489.0)	450.5 (381.9)	53.0 (−131.6 to 237.6)*
	Median [IQR]	545.7 [100.6 to 942.1]	325.6 [71.0 to 745.0]	
28+0 to 31+6	Mean (SD)	609.4 (368.5)	488.9 (312.8)	100.0 (−99.3 to 299.4)*
	Median [IQR]	516.0 [303.7 to 847.0]	490.6 [243 to 677.8]	
*Missing (n)*		*16*	*8*	
Exclusive breastmilk^b^ at 36 weeks’ PMA, *n* (%)				
23+0 to 27+6		18 (58)	18 (62)	
28+0 to 31+6		19 (70)	19 (73)	
*Missing*		*10*	*9*	
Any breastmilk^c^ at 36 weeks’ PMA, *n* (%)				
23+0 to 27+6		26 (84)	23 (79)	
28+0 to 31+6		25 (93)	24 (92)	
*Missing*		*10*	*9*	
**Birth intention to give exclusive breastmilk at discharge**^**d**^				
*Missing data for breastmilk intention (n)*		*9*	*2*	
24-h milk weight (g)^a^				
Yes	Mean (SD)	622.3 (485.8)	520.5 (370.3)	
	Median [IQR]	572.2 [198.6 to 1041.9]	502.6 [211.4 to 810.1]	
No/unsure	Mean (SD)	539.2 (288.6)	355.8 (254.6)	
	Median [IQR]	521.4 [340.9 to 717.5]	321.7 [157.1 to 652.8]	
*Missing (n)*		*7*	*7*	
Exclusive breastmilk^b^ at 36 weeks’ PMA, *n* (%)				
Yes		27 (69)	28 (74)	
No/unsure		8 (57)	8 (50)	
*Missing*		*6*	*8*	
Any breastmilk^c^ at 36 weeks’ PMA, *n* (%)				
Yes		34 (87)	33 (87)	
No/unsure		13 (93)	13 (81)	
*Missing*		*6*	*8*	
**Multiple birth**				
24-h milk weight (g)^a^				
Singleton	Mean (SD)	518.8 (377.8)	466.4 (356.2)	
	Median [IQR]	481.4 [198.9 to 838.2]	397.8 [162.1 to 740.2]	
Multiple birth	Mean (SD)	1025.2 (494.7)	475.2 (333.7)	
	Median [IQR]	1118.0 [640.0 to 1221.2]	399.7 [279.2 to 673.6]	
*Missing (n)*		*16*	*8*	
Exclusive breastmilk^b^ at 36 weeks’ PMA, *n* (%)				
Singleton		31 (63)	32 (68)	
Multiple birth		6 (67)	5 (63)	
*Missing*		*10*	*9*	
Any breastmilk^c^ at 36 weeks’ PMA, *n* (%)				
Singleton		42 (86)	40 (85)	
Multiple birth		9 (100)	7 (88)	
*Missing*		*10*	*9*	
**Adherence group**				
24-h milk weight (g)^a^				
Higher adherence (≥3 times/day)	Mean (SD)	703.1 (395.3)		
	Median [IQR]	778 [346 to 1010]		
Lower adherence (<3 times/day)	Mean (SD)	524.6 (449.5)		
	Median [IQR]	391 [170 to 687]		
*Missing (n)*		*16*		
**Perception of relaxation group**				
* Missing data for perception of relaxation*		*17*		
24-h milk weight (g)^a^				
Perceived intervention as relaxing (*n* = 40)	Mean (SD)	609.8 (445.2)		
	Median [IQR]	571 [255 to 903]		
Did not perceive intervention as relaxing (*n* = 10)	Mean (SD)	625.7 (400.3)		
	Median [IQR]	599 [262 to 1010]		
*Missing (n)*		*1*		
**Perception of recording group**				
*Missing data for perception of recording*		*17*		
24-h milk weight (g)^a^				
Liked intervention (*n* = 37)	Mean (SD)	659.2 (447.5)		
	Median [IQR]	684 [318 to 946]		
Disliked intervention (or neutral; *n* = 13)	Mean (SD)	481.5 (372.3)		
	Median [IQR]	478 [104 to 778]		
*Missing (n)*		*1*		

*PMA* postmenstrual age, *CI* confidence interval, *SD* standard deviation, *IQR* interquartile range, *NA* not applicable as no statistical inference pre-planned.

*Interaction *p*-value = 0.73. Missing observations are excluded from percentage denominators.

^a^At any submitted log on day 4, 14 or 21. Adjusted for gestational age at birth, recruitment centre, multiple birth and measurement day.

^b^Exclusive breastmilk is defined as no infant formula used in the last 24 h. If two babies, then no infant formula in the last 24 h for either baby.

^c^Any breastmilk is defined as any use of breastmilk in the last 24 h, either direct breastfeeding or as expressed milk. If two babies, then any breastmilk for either baby.

^d^Defined as “your baby would be drinking only your breastmilk and no infant formula”.

The higher adherence group (listening at least three times a day, *n* = 21) had a primary outcome of 703.1 g (SD 395.3) compared to 524.6 g (SD 449.5) in the lower adherence group (*n* = 31; Table 3). There was no difference in primary outcome according to perceived relaxation (Table 3). No baseline or expressing-related variables were significantly associated with adherence (Supplementary Table 5) or perceived relaxation (data not shown).

There were no differences in baseline characteristics for participants with present or absent primary outcome data (Supplementary Table 6). In addition to the multiple imputation approach to missing data reported in Supplementary Table 4, a pattern mixture model was performed (Supplementary Fig. 1). The adjusted mean difference under the modelled conditions ranged from 27.2 g (−108.2 to 162.6 g) to 122.2 g (−13.2 to 257.6 g).

There was no evidence of a difference in secondary outcomes or process indicators (Table 2 and Supplementary Table 7). With reference to the threshold for suspicion of clinical anxiety, 58% (25/43) of the intervention group and 56% (23/41) of the control group fulfilled these criteria on day 21 after birth. For suspicion of clinically significant post-traumatic stress reactions, 14% (6/44) of the intervention group and 23% (10/44) of the control group fulfilled these criteria on day 21 after birth.

**Fig. 2:**
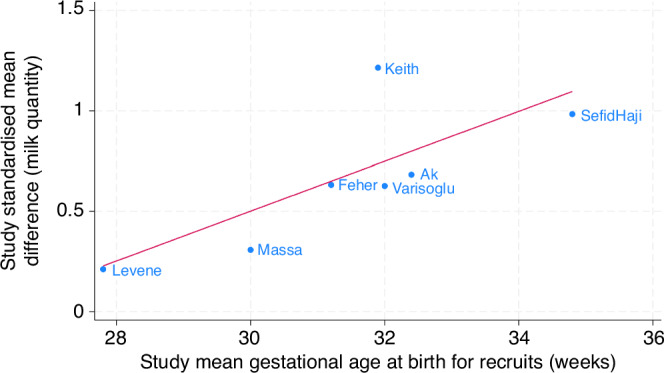
Meta-regression. This shows the relationship of expressed mothers’ own milk quantity by gestational age of birth, with each study as a named datapoint (hospital-based studies only).

Post-hoc meta-regression combined this study with six others^16,20,24–27^ recently identified in the meta-analysis,^15^ in relation to the outcome of milk quantity (Fig. 2). For an increase in the mean gestational age at birth of trial recruits by a week, the standardised mean difference for milk quantity increased by 0.12 (95% CI 0.01 to 0.26). Gestational age explained 69% of between-study variance in the milk quantity effect estimate (R^2^).

## Discussion

This trial showed no evidence of an effect of the relaxation and lactation-specific visualisation recording on lactation, anxiety or post-traumatic stress, despite high adherence. The majority of women liked the intervention and found it relaxing.

Participants with higher reported adherence to the intervention had more MOM than those with lower adherence. Higher adherence was not associated with any other variables that might suggest this was due to confounding, but this cannot be excluded as this is a non-randomised comparison. This increased effect in more adherent participants has been demonstrated in several other trials.^16,25^

### Setting the trial results in context

The lack of increase in milk quantity seen in this trial is in conflict with recent meta-analysis.^15^ Post-hoc meta-regression has suggested a new hypothesis for this disparity; that the effect of relaxation on milk quantity may be smaller for mothers who have given birth at lower gestation. As noted in the introduction, these women have a higher level of physiological challenge to establish full lactation.

The direction of subgroup effects seen in this report would be consistent with this hypothesis, whereby the effect size was larger for mothers of very preterm compared to extremely preterm infants, but this was not statistically significant, and the study was not powered to detect such a relationship.

Meta-analysis of previous studies^15^ showed a likely small reduction in maternal anxiety with relaxation provision, with high statistical heterogeneity. Two out of three neonatal unit studies showed a reduction in anxiety within the meta-analysis.^27,28^ The neonatal unit study showing no difference in anxiety with relaxation (a mindfulness app) had the highest level of overall reported anxiety and the lowest mean birth gestational age of the three.^16^ Our population also had a high level of baseline anxiety and an even more extreme level of prematurity. This may suggest that relaxation is less effective in reducing anxiety when baseline anxiety is high, but there are insufficient studies to explore this more formally.

No previous studies have reported on post-traumatic stress after the use of a lactation-focused relaxation/visualisation. There was no significant change in the PCL-5. Some participants subjectively related the use of the recording to reframing their experience of birth trauma.

### Strengths and limitations

The key limitation of the study is the moderate level of missing data for some outcomes, particularly mental health outcomes. In addition, there was an unequal pattern of missing data between allocation arms. Without further exploration, this would put the results at high risk of bias. However, one strength of the study was the close attention paid to identifying potential bias and minimising its impact on the study conclusions, through statistical adjustment, sensitivity analysis and modelling approaches to missing data. There was a large reduction in the primary outcome effect estimate after adjustment, and when using sensitivity analyses designed to address potential bias from study design and missing data, demonstrating the need for this approach. In particular, pattern mixture modelling shows that even a large amount of intervention-arm-specific bias in missing data would not have changed the conclusions of our analysis. Where statistical analysis demonstrates that missing data would not change the conclusion of the analysis, these can be considered as presenting a low risk of bias.^29^

The lack of blinding is an unavoidable potential limitation. As many participants were in frequent contact with each other it was not possible to use blinding through partial deception, as used in some prior studies.^30–32^ Any effort to produce a control recording was thought to be at high risk of either producing relaxation by another means (for example white noise or a time countdown), or causing stress to participants, which would be unethical.

A further limitation was the fact that participants weighed their own milk, rather than this being done by researchers. This was explicitly recommended by parent collaborators during study design to minimise any possibility that freshly expressed milk might not be immediately available to feed the infant and to minimise participant perception of judgement and pressure surrounding the sensitive topic of milk quantity. This is discussed in more detail in a previous publication.^21^

A further strength is external validity. The study was multi-centre and participants were diverse in socioeconomic and ethnic background. This study recruited participants with a higher level of prematurity than any other study^15^ of relaxation and lactation, which is important to increase the applicability of meta-analysis findings to this high-stress, high-risk population.

Participant comments suggested that adherence and enjoyment were affected by the lack of variety in the relaxation material. The increased loss to follow-up in the intervention group and the reduced number of multiparous women choosing to take part suggests that the intervention might have been burdensome or perceived as potentially burdensome, to some. In addition, 12% of the intervention group reported disliking the intervention, and comments showed that some found it a chore or felt pressure to listen. These factors may have impaired adherence and reflect that not all parents find relaxation acceptable. Increasing the variety of relaxation material available could increase acceptability and potentially adherence.

The level of relaxation use in the control group was relatively high. If this were related to the trial context (for example, due to receiving information about relaxation through the consent process), then this would pose a risk of bias to the results. However, some background use of relaxation is expected; a previous study of women of child-bearing age interested in a relaxation RCT reported that 53% regularly used relaxation prior to recruitment.^33^ The rate of relaxation use in the control group is therefore more likely to be a feature of the real-world baseline environment than trial-related “contamination”.

When considering the variability of real-life relaxation use in both allocation arms, it is useful to consider the concept of the ‘estimand’, which is a specific definition of the estimated treatment effect, considering postrandomisation events.^34^ The estimand for this study relates to the provision of a specific relaxation recording (correct allocation received by all participants), rather than the parent’s actual use of relaxation materials (variable between participants in both allocation arms). This does not make the estimand less valid but does mean that the results are more useful for clinical teams (“Is it worth recommending this relaxation intervention to families?”) than for parental decision-making (“What is the likely effect of using any relaxation material in a particular way?”).

There was no evidence that participant-reported outcomes were influenced by a belief in the intervention or desire to please the research team despite the unavoidable unmasked nature of the trial. Mental health scores would be most vulnerable to this possibility due to their self-reported nature and showed no signal of treatment effect. Similarly, there was no signal of difference in expressing behaviours such as duration or frequency of expressing.

## Conclusion

Although this study did not show evidence of improvement in lactation or mental health outcomes, participants predominantly enjoyed the relaxation intervention and perceived it as relaxing. There is a low cost to health service resource and a low risk of harm. However, some found the intervention unpleasant and burdensome, demonstrating that there may be a negative impact on individuals and opportunity cost to mothers.

Using the results of this study alone, this intervention cannot be recommended in the context of very preterm birth but can be offered with a discussion of potential harms and benefits. A small increase in milk quantity may nevertheless have clinical importance in this population, which this study was not powered to detect.

## Supplementary information

Reporting checklist

Supplementary Information

Supplemental Audio File 1

## Data Availability

De-identified individual participant data (including data dictionaries) may be made available with the publication, in addition to study protocols, the statistical analysis plan, and the informed consent form. A review of all requests for sharing of the study data will take place as described in the National Perinatal Epidemiology Unit Standard Operating Procedures on data sharing. If agreed, any sharing of the data collected during the study must be in accordance with the Nuffield Department of Population Health and University of Oxford policies. Instructions on requesting access are provided here: https://www.npeu.ox.ac.uk/ctu/data-sharing.
