# Supplementary material for: Lactation-focused audio relaxation versus standard care for mothers of very preterm infants (the EXPRESS randomised clinical trial)
Source: Pediatr Res. 2024 Sep 25;97(6):2035–44. doi: 10.1038/s41390-024-03577-7 (PMC12122367; doi:10.1038/s41390-024-03577-7)
Supplement: Supplementary file 2 — Supplementary Information [file 41390_2024_3577_MOESM2_ESM.pdf]

List of Supplementary material:

Supplemental Table 1: Comparison of the population eligible for screening with the trial participants recruited
Supplemental Table 2: Participant adherence and contamination, and data quality
Supplemental Table 3: Qualitative comments about the intervention
Supplemental Table 4: Sensitivity analyses for the primary outcome
Supplemental Table 5: Comparison of baseline data and process indicators between participants with higher and lower adherence
Supplemental Table 6: Comparison of baseline data between participants with available and missing data for primary outcome
Supplemental Table 7: Day 14 process indicators

Supplemental Figure 1: Pattern Mixture Modelling for missing data for the primary outcome

Supplemental Audio File 1 (separate file): Audio file of trial intervention relaxation

This is a 12 minute voice recording for parents who are expressing milk in the neonatal unit context. It contains a guided muscle relaxation, breathing exercises, guided imagery and lactation visualisation. It was modified from an existing recording under license from the original author (Sheri Menelli). The voice recording was made by Lucy Livesey.

Supplemental Table 1: Comparison of the population eligible for screening with the trial participants recruited

	**Trial participants (n = 132)**	**Population eligible for screening[a] (n = 381)**
**Gestational age at birth, n (%)**		
**23+0 to 27+6**	71 (53.8)	203 (53.3)
**28+0 to 31+6**	61 (46.2)	178 (46.7)
Missing	*0*	*0*
**Primiparous, n (%)**	74 (59.7)	165 (48.5)
Missing	*8*	*41*
**Multiple pregnancy, n (%)**	20 (15.2)	54 (17.0)
Missing	*0*	*64*
**Mode of delivery, n (%)**		
**Vaginal birth**	57 (43.2)	157 (42.3)
**Caesarean birth**	75 (56.8)	214 (57.7)
Missing	*0*	*10*
**Participant age (years), mean (SD)**	32.8 (6.3)	32.3 (6.8)
**Median [IQR]**	32.8 [29.6 to 35.6]	32.8 [28.0 – 35.5]
**(Min to max)**	(19 to 54)	(17 to 54)
Missing	*0*	*6*

[a]Anonymized data extracted from prospectively entered clinical database (Badgernet, Clevermed). Information related to twin one is used for multiple births, to represent the mother. This population includes the trial participants, mothers who declined participation, mothers who were not eligible to consent after full screening and mothers who were not approached but fell into the appropriate gestational age group. Missing observations are excluded from percentage denominators

Supplemental Table 2: Participant adherence and contamination, and data quality

	**Relaxation group (n = 68)**	**Control group (n = 64)**
**Listened to/practiced non-intervention relaxation techniques by day 21, n (%)**		
Daily or more	3 (7)	3 (7)
More than once a week, less than once a day	8 (19)	6 (14)
Once a week or less	16 (38)	2 (5)
Not at all	15 (36)	33 (75)
Missing	*26*	*20*
**Frequency of listening to intervention recording (per day)**		
**Day 4**		
Mean (SD)	3.8 (3.3)	-
Median [IQR]	3 [1 to 6]	-
Missing	*25*	-
**Day 14**		
Mean (SD)	3.5 (3.0)	-
Median [IQR]	3 [1 to 5]	-
Missing	*26*	-
**Day 21**		
Mean (SD)	3.0 (2.5)	-
Median [IQR]	3 [1 to 5]	-
Missing	*26*	-
**Ever reported listening to recording, n (%)**	51 (98)	-
Missing	*16*	-
**Perception of intervention recording on day 21, n (%)**		
Like/very much like	25 (60)	-
Neutral	12 (29)	-
Dislike/very much dislike	5 (12)	-
Missing	*26*	-
**Perception of effect of intervention recording on day 21, n (%)**		
More relaxed/much more relaxed	32 (76)	-
Neutral	6 (14)	-
Less relaxed/much less relaxed	4 (10)	-
Missing	*26*	-
**Data available for consideration for primary outcome, n (%):**		
Day 4	49 (72)	52 (81)
Day 14	45 (66)	45 (70)
Day 21	47 (69)	43 (67)
**Timepoint used for primary outcome, n (%):**		
Day 4	8 (15)	14 (25)
Day 14	11 (21)	15 (27)
Day 21	33 (64)	27 (48)
No data available	*16*	*8*

	**Relaxation group (n = 68)**	**Control group (n = 64)**
**Logs submitted inside the scheduled window[a]. n/N (% of submitted logs):**		
Day 4	50/51 (98)	52/53 (98)
Day 14	46/46 (100)	45/45 (100)
Day 21	48/48 (100)	43/43 (100)
**Logs with erroneous data removed n/N (% of submitted logs):**		
Day 4	3/51 (6)	5/53 (9)
Day 14	1/46 (2)	3/45 (7)
Day 21	4/48 (8)	2/43 (5)
**Logs with <4 expressing sessions recorded. n/N (% of submitted logs):**		
Day 4	10/51 (20)	13/53 (25)
Day 14	7/46 (15)	3/45 (7)
Day 21	6/48 (13)	4/43 (9)

SD = standard deviation; IQR = interquartile range. Missing observations are excluded from percentage denominators

[a] *The scheduled window is 48 hours – for example a day 4 log that is started on day 4, day 5 or before 10am on day 6 is inside the schedule window*

Supplemental Table 3: Qualitative comments about the intervention

Participant	Day submitted	Response to the question "Do you have any comments about the study recording?"
2	Day 21	I have started to find I stop 'listening' but enjoy how the voice washes over me. I still find the comment about learning to be a mother irritates me and the mention of a to-do list makes me think about everything I need to do when I haven't before!
4	Day 21	Find it difficult to listen to the recording as in the hospital so much
11	Day 4	The recording helps to relax when expressing in different environments. It allows me to block out the noise and distractions with the breathing exercises. I focus less on the amount I am expressing and I have been able to express for longer. The recording could be a bit longer but I can always restart.
16	Day 21	Nothing other than it would be nice to have some variety.
23	Day 14	It's good, I just wouldn't listen to it all the time as it can get quite repetitive
26	Day 21	As I have heard the study recording quite a few times now, it can feel a bit repetitive. I feel quite relaxed when I begin listening to it, but as I get towards the middle/end I find myself getting more distracted now.
28	Day 21	Needed to be longer personally
33	Day 4	It could be a little longer
34	Day 4	I'd like it if it was a bit longer
39	Day 14	If [baby] is having a good day the recording is brilliant i can actually pay attention to it when we have a day which isnt as good i find it hard to concentrate on it, it becomes more of a chore than a relaxation method.
41	Day 21	Finding time to remember to listen is tricky
42	Day 4	It's helping calm me whilst I feed
47	Day 21	I feel pressure about listening to the recording, and I don't really like listening to it (sorry)
53	Day 4	This study has really helped keep me calm and keep my mind off things
54	Day 21	It is boring to listen to the same thing over and over again.
59	Day 14	Struggle at times to find quite [quiet] space to express, especially at hospital due to either bedside expressing or room with curtain separating from kitchen area.
60	Day 4	Good
62	Day 4	I think I would prefer calming music rather that someone talking. I feel this would telex [relax] me a lot more
72	Day 21	I think the recording is very helpful
87	Day 21	It could be longer
89	Day 21	I would like to do it with some background music or natural world sounds
91	Day 14	So far I think the recording could be helpful sometimes but for me I feel like I express more milk when I am with my baby or I am looking at a picture of him. I feel less stressed. The recording helps you relax but I don't know if it affects how much I'm expressing.

Participant	Day submitted	Response to the question "Do you have any comments about the study recording?"
98	Day 21	Excellent
106	Day 21	Found it a bit harder to focus on this week, perhaps because I've now listened so many times! On stressful days it definitely makes me more calm. It's a good length - enough to relax but not too long, once it finishes I pay a bit more attention to expressing & any areas that need a massage etc.
111	Day 21	Nice relaxing voice
112	Day 14	The recording is still helpful. I believe because my birth and recovery has felt rather traumatic, the recording reminds me to focus on the things that I can do right now and being present while I'm expressing focusing on the love I have for my child as opposed to all the other unsavoury bits.
117	Day 14	It's good
118	Day 14	It's good for relaxing
121	Day 21	It really good to listen to because it makes you relaxed.
126	Day 21	I still find it difficult to fully listen to the recording - my mind flicks to other thoughts frequently but i do find my mind is always back on the recording by the end (e.g. for the count down). After 3 weeks of listening it would be good to have a slightly different version of it - perhaps versions that focus on different aspects of relaxation combined with thoughts of your baby (e.g. more on breathing exercises, more on relaxing body parts) or versions that you could listen to whilst driving to the hospital as even though you are not pumping at the time it helps get through a more stressful part of the day. This week ive had quite a few issues with blocked ducts so it would be good to have a part at the start that perhaps focuses on massage – becayse [because] i never make time to do this before pumping.
130	Day 4	The recording is relaxing - but listening to the same recording every time is very repetitive and I think would put me off long term.
132	Day 14	I haven't used the recording as it makes no sense too and have no quiet time

Where a participant submitted more than one comment, the latest chronological comment is reported. Presented as written by participants. Infant name replaced by [baby]. Suggested clarifications in square brackets if required

Supplemental Table 4: Sensitivity analyses for the primary outcome

	Relaxation Group (n = 68)	Control Group (n = 64)	Unadjusted MD (95% CI)	Adjusted MD (95% CI)	Adjusted p value
**Primary Outcome: Highest 24-hour milk weight on day 4, 14 or 21 (grams)**					
**Mean (SD)**	596.7 (433.6)	467.7 (350.2)	129.1 (-20.8 to 278.9)	73.9 (-61.7 to 209.5)	0.28
**Median [IQR]**	521.4 [254.6 to 902.7]	397.8 [176.6 to 719.2]			
Missing	*16*	*8*			
**Sensitivity analysis 1: Using only day 21 milk weight (grams)**					
**Mean (SD)**	596.4 (434.8)	521.1 (325.9)	75.3 (-86.9 to 237.5)	73.0 (-91.9 to 237.9)	0.38
**Median [IQR]**	547 [247.5 to 938.4]	473.8 [243 to 735.3]			
Excluded versus PO	*5*	*13*			
**Sensitivity Analysis 2: Using multiple imputation for all missing 24-hour weights (grams)**					
**Mean (SD)**	612.1 (433.7)	533.9 (369.2)	78.2 (-63.5 to 219.9)	71.3 (-64.3 to 207.0)	0.30
Added versus PO	*16*	*8*			
**Sensitivity analysis 3: Excluding day 4 milk weight (grams)**					
**Mean (SD)**	603.2 (437.2)	529.4 (331.7)	73.8 (-83.3 to 231.0)	46.3 (-108.9 to 201.5)	0.56
**Median [IQR]**	521.4 [247.5 to 938.4]	483.2 [269.8 to 783.3]			
Excluded versus PO	*2*	*9*			
**Sensitivity analysis 4: Excluding all expressing logs with less than four sessions logged (grams)**					
**Mean (SD)**	666.3 (420.8)	510.1 (340.0)	156.3 (-2.9 to 315.5)	108.4 (-45.9 to 262.8)	0.17
**Median [IQR]**	684.3 [336.2 to 945.7]	482.2 [236 to 745]			
Excluded versus PO	*7*	*10*			
**Sensitivity analysis 5: Excluding logs submitted >48 hours after scheduled timepoint (grams)**					
**Mean (SD)**	596.7 (433.6)	465.7 (350.0)	131.0 (-18.9 to 280.8)	72.9 (-64.3 to 210.1)	0.29
**Median [IQR]**	521.4 [254.6 to 902.7]	397.8 [176.6 to 719.2]			
Excluded versus PO	*0*	*0*			

CI = confidence interval; SD = standard deviation; IQR = interquartile range. PO = primary outcome. Missing observations are excluded from percentage denominators

[a]At any submitted log on day 4, 14 or 21. Adjusted for gestational age at birth, recruitment centre, multiple birth and measurement day.

Supplemental Table 5: Comparison of baseline data and process indicators between participants with higher and lower adherence

	**Higher adherence (n = 21)**	**Lower adherence (n = 31)**
**Participant ethnic background[a], n (%)**		
Asian or Asian British	4 (19)	3 (10)
Black, African, Black British or Caribbean	2 (10)	8 (26)
White or White British	14 (67)	18 (58)
Other	1 (5)	1 (3)
Prefer not to say	0 (0)	1 (3)
**Participant age (years), mean (SD)**	33.3 (4.5)	33.0 (6.7)
**Index of Multiple Deprivation quintile[b], n (%)**		
1 (Most deprived)	4 (19)	8 (26)
2	4 (19)	3 (10)
3	3 (14)	5 (16)
4	6 (29)	3 (10)
5 (Least deprived)	4 (19)	12 (39)
**Age at leaving full-time education, n (%)**		
16 years old or less	1 (5)	2 (6)
17 or 18 years old	6 (29)	4 (13)
19 years old or more	12 (57)	22 (71)
Still in full time education	2 (10)	2 (6)
Prefer not to say	0 (0)	1 (3)
**Lives with a partner, n (%)**	19 (90)	29 (94)
**Current smoker, n (%)**	0 (0)	4 (13)
**Time from birth to first expression of milk (hours), median [IQR]**	5 [3 – 12]	6 [3 – 15]
**Intention for exclusive breastmilk at time of discharge[c], n (%)**	14 (67)	22 (71)
**Spielberger State-Trait Anxiety Index[d] score at randomisation, mean (SD)**	55.2 (13.5)	53.4 (14.9)
Score >40	16 (80)	24 (83)
Missing	*2*	*1*
**Mode of delivery, n (%)**		
Vaginal birth	5 (24)	10 (32)
Caesarean birth	16 (76)	21 (68)
**Multiple pregnancy, n (%)**	2 (10)	6 (19)
**Primiparous, n (%)**	10 (48)	19 (61)
**Recruiting centre, n (%)**		
1	12 (57)	19 (61)
2	3 (14)	9 (29)
3	2 (10)	2 (6)
4	4 (19)	1 (3)
**Gestational age at birth (weeks), n (%)**		
Median [IQR]	28.7 [27.1 – 30.6]	27.0 [25.4 – 29.0]
23 to < 26 weeks	5 (24)	9 (29)
26 to < 28 weeks	2 (10)	12 (39)
28 to < 30 weeks	6 (29)	6 (19)
30 to < 32 weeks	8 (38)	4 (13)

	**Higher adherence (n = 21)**	**Lower adherence (n = 31)**
**Ventilated at randomisation (one or both infants), n (%)**	10 (48)	7 (23)
**Expressing frequency on day 4**	6 [4 – 8]	5 [4 – 7]
Missing	*0*	*2*
**Expressing frequency on day 14**	7 [5 – 7]	6 [4-8]
Missing	*1*	*5*
**Expressing frequency on day 21**	7 [5 – 7]	6 [4.5 – 7]
Missing	*1*	*3*
**Duration of expressing on day 4**	108 [90 – 170]	135 [60 – 210]
Missing	*0*	*2*
**Duration of expressing on day 14**	141.5 [102.5 – 197.5]	168 [75 – 194]
Missing	*1*	*5*
**Duration of expressing on day 21**	141 [87.5 – 202.5]	159 [107.5 – 194.5]
Missing	*1*	*3*

Higher adherence is listening to the recording at least three times a day on the last reported timepoint. SD = standard deviation; IQR = interquartile range. Missing observations are excluded from percentage denominators

[a]*Categories defined by the United Kingdom Office of National Statistics*

[b]*England postcodes are assigned an Index of Multiple Deprivation quintile according to multiple factors associated with area level deprivation*

[c]*Defined as "your baby would be drinking only your breastmilk and no infant formula"*

[d]*Six item version, transformed to an equivalent score to 20 item original. Score range is 20 to 80, higher score indicates more anxiety*

Supplemental Table 6: Comparison of baseline data between participants with available and missing data for primary outcome

	**Primary Outcome available (n = 108)**	**Primary Outcome missing (n = 24)**
**Participant ethnic background[a], n (%)**		
Asian or Asian British	19 (18)	3 (21)
Black, African, Black British or Caribbean	20 (19)	1 (7)
White or White British	62 (58)	10 (71)
Mixed or multiple ethnic groups	3 (3)	0
Other	2 (2)	0
Prefer not to say	1 (1)	0
Missing	*1*	*10*
**Participant age (years), mean (SD)**	33.0 (6.0)	32.0 (7.7)
Median [IQR]	33.3 [29.9 to 35.8]	32.4 [26.1 to 35.1]
(Min to max)	(21 to 55)	(19 to 50)
**Index of Multiple Deprivation quintile[b], n (%)**		
1 (Most deprived)	19 (18)	5 (22)
2	23 (21)	3 (13)
3	18 (17)	4 (17)
4	22 (20)	9 (39)
5 (Least deprived)	26 (24)	2 (9)
Missing	*0*	*1*
**Age at leaving full-time education, n (%)**		
16 years old or less	11 (10)	3 (21)
17 or 18 years old	21 (20)	3 (21)
19 years old or more	69 (65)	8 (57)
Still in full time education	5 (5)	0
Prefer not to say	1 (1)	0
Missing	*1*	*10*
**Lives with a partner, n (%)**	96 (90)	12 (86)
Missing	*1*	*10*
**Current smoker, n (%)**	7 (7)	1 (7)
Missing	*1*	*10*
**Intensive care in first 48 hours after birth, n (%)**	1 (1)	0 (0)
**Time from birth to first expression of milk (hours), median [IQR]**	6 [3 to 12]	6.5 [2 to 12]
(Min to max)	(0 to 96)	(1 to 72)
Missing	*2*	*10*
**Intention for exclusive breastmilk at time of discharge[c], n (%)**	76 (71)	9 (64)
Missing	*1*	*10*
**Spielberger State-Trait Anxiety Index[d] score at randomisation, mean (SD)**	52.1 (14.3)	49.8 (14.2)
Median [IQR]	5 [40 to 63]	47 [40 to 54]
(Min to max)	(20 to 80)	(33 to 77)
Score >40	73 (75)	7 (58)
Missing	*10*	*12*
**Mode of delivery, n (%)**		

	**Primary Outcome available (n = 108)**	**Primary Outcome missing (n = 24)**
Vaginal birth	44 (41)	13 (54)
Caesarean birth	64 (59)	11 (46)
**Multiple pregnancy, n (%)**	16 (15)	4 (17)
**Primiparous, n (%)**	64 (60)	10 (59)
Missing	*1*	*7*
**Recruiting centre, n (%)**		
1	65 (60)	12 (50)
2	25 (23)	7 (29)
3	8 (7)	2 (8)
4	10 (9)	3 (13)
**Gestational age at birth (weeks), n (%)**		
Median [IQR]	27.6 [25.9 to 29.9]	27.9 [26.4 to 30.1]
(Min to Max)	(23 to 32)	(24 to 32)
23 to < 26 weeks	28 (26)	6 (25)
26 to < 28 weeks	31 (29)	6 (25)
28 to < 30 weeks	24 (22)	5 (21)
30 to < 32 weeks	25 (23)	7 (29)
**Ventilated at randomisation (one or both infants), n (%)**	39 (36)	8 (33)

SD = standard deviation; IQR = interquartile range. Missing observations are excluded from percentage denominators

[a]*Categories defined by the United Kingdom Office of National Statistics*

[b]*England postcodes are assigned an Index of Multiple Deprivation quintile according to multiple factors associated with area level deprivation*

[c]*Defined as "your baby would be drinking only your breastmilk and no infant formula"*

[d]*Six item version, transformed to an equivalent score to 20 item original. Score range is 20 to 80, higher score indicates more anxiety*

Supplemental Table 7: Day 14 process indicators

	**Relaxation group (n = 68)**	**Control group (n = 64)**
**Time spent in skin to skin contact (hours)[b], mean (SD)**	2.0 (1.7)	1.7 (1.6)
Median [IQR]	2 [0 to 3]	1.8 [0 to 2.4]
Min to max	(0 to 6)	(0 to 7)
Missing	*25*	*20*
**Expressing episodes in 24 hours[c], mean (SD)**	5.9 (2.2)	6.0 (1.6)
Median [IQR]	6 [5 to 7]	6 [5 to 7]
Min to max	(0 to 11)	(1 to 9)
No longer lactating	1 (2.0)	0 (0.0)
No longer expressing; solely breastfeeding	0 (0.0)	0 (0.0)
Missing	*22*	*19*
**Time spent expressing (hours), mean (SD)**	153.7 (80.6)	169.4 (76.6)
Median [IQR]	160 [100 to 195]	161 [120 to 210]
Min to max	(0 to 430)	(30 to 390)
Missing	*22*	*19*
**Perception of low milk supply, n (%)**	12 (28)	19 (43)
Missing	*25*	*20*
**Direct breastfeeds[a], mean (SD)**	0.1 (0.5)	0.0 (0.2)
Median [IQR]	0 [0 to 0]	0 [0 to 0]
Min to max	(0 to 2)	(0 to 1)
Missing	*41*	*30*

Missing observations are excluded from percentage denominators

[a] *A direct breastfeed is defined as an episode at the breast where the mother felt the baby was sucking and swallowing some milk. If the baby is offered both breasts at a single episode this is one breastfeed. If the baby stops sucking or swallowing for 30 minutes or more, then any further feeding is a new breastfeed. If two babies, then the sum of all episodes with both babies.*

[b] *If two babies, then the sum of time spent in skin to skin contact with both babies*

[c] *An expressing episode is defined as where two breasts are expressed simultaneously or where two breasts are expressed sequentially, with the start time of the second breast expression within 10 minutes of the end time of the first breast expression or where one breast is expressed alone*

Supplemental Figure 1: Pattern Mixture Modelling for missing data for the primary outcome

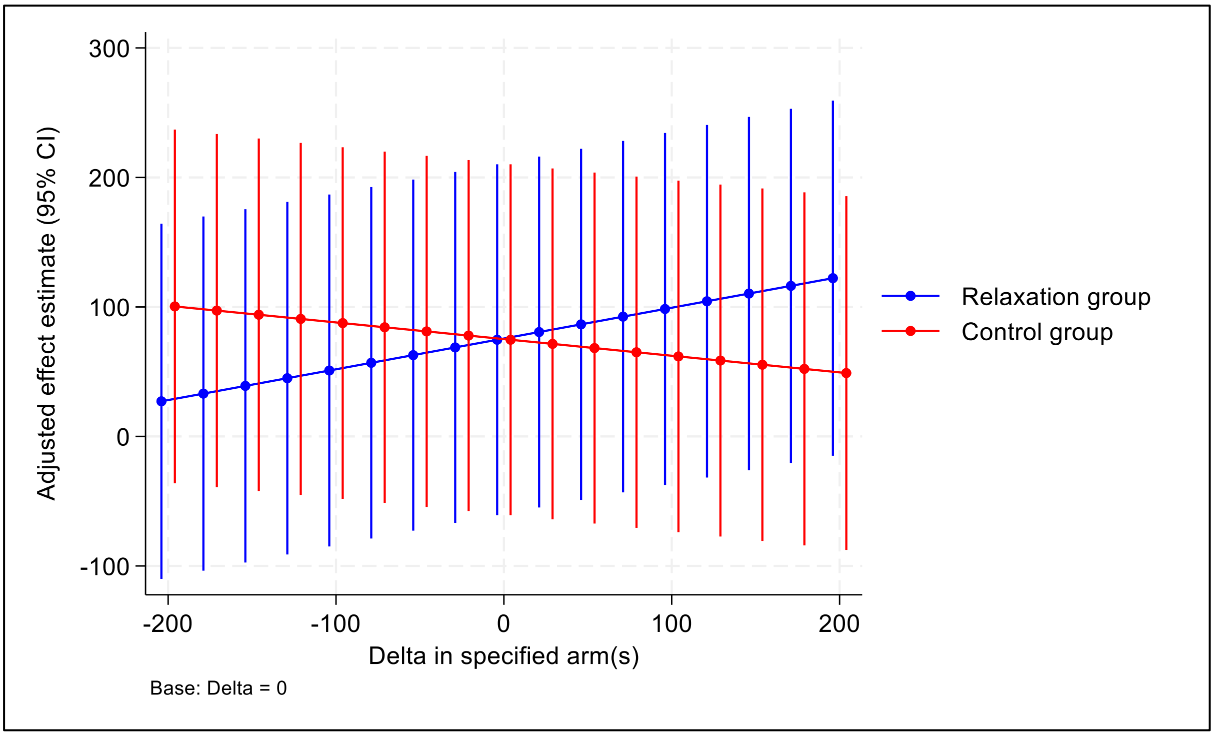

A pattern mixture model is used on the adjusted primary outcome analysis with a range of effect sizes (delta) from -200g to +200g. This means that where the primary outcome is missing in the specified arm, it is imputed as the average value plus the delta value, to assess the effect of missing values being different to recorded values by up to 200g. Missing data in the other arm is imputed as the average value.
